# Proteomic signature associated with chronic kidney disease (CKD) progression identified by data-independent acquisition mass spectrometry

**DOI:** 10.1186/s12014-023-09405-0

**Published:** 2023-04-20

**Authors:** Carlos R. Ramírez Medina, Ibrahim Ali, Ivona Baricevic-Jones, Aghogho Odudu, Moin A. Saleem, Anthony D. Whetton, Philip A. Kalra, Nophar Geifman

**Affiliations:** 1 Stoller Biomarker Discovery Centre, Faculty of Biology, Medicine and Health, The University of Manchester, Manchester, UK; 2 Salford Royal Hospital, Northern Care Alliance NHS Foundation Trust, Salford, UK; 3 Division of Cardiovascular Sciences, The University of Manchester, Manchester, UK; 4 Bristol Renal and Children’s Renal Unit, Bristol Medical School, University of Bristol, Bristol, UK; 5 School of Veterinary Medicine, Faculty of Health and Medical Sciences, University of Surrey, Guildford, GU2 7XH UK; 6 School of Health Sciences, Faculty of Health and Medical Sciences, University of Surrey, Guildford, GU2 7XH UK

**Keywords:** Chronic kidney disease (CKD) Progression, Proteomics, SWATH-MS, Complement cascade pathway, Proteasome pathway, Biomarkers

## Abstract

**Background:**

Halting progression of chronic kidney disease (CKD) to established end stage kidney disease is a major goal of global health research. The mechanism of CKD progression involves pro-inflammatory, pro-fibrotic, and vascular pathways, but pathophysiological differentiation is currently lacking.

**Methods:**

Plasma samples of 414 non-dialysis CKD patients, 170 fast progressors (with ∂ eGFR-3 ml/min/1.73 m^2^/year or worse) and 244 stable patients (∂ eGFR of − 0.5 to + 1 ml/min/1.73 m^2^/year) with a broad range of kidney disease aetiologies, were obtained and interrogated for proteomic signals with SWATH-MS. We applied a machine learning approach to feature selection of proteins quantifiable in at least 20% of the samples, using the Boruta algorithm. Biological pathways enriched by these proteins were identified using ClueGo pathway analyses.

**Results:**

The resulting digitised proteomic maps inclusive of 626 proteins were investigated in tandem with available clinical data to identify biomarkers of progression. The machine learning model using Boruta Feature Selection identified 25 biomarkers as being important to progression type classification (Area Under the Curve = 0.81, Accuracy = 0.72). Our functional enrichment analysis revealed associations with the complement cascade pathway, which is relevant to CKD as the kidney is particularly vulnerable to complement overactivation. This provides further evidence to target complement inhibition as a potential approach to modulating the progression of diabetic nephropathy. Proteins involved in the ubiquitin–proteasome pathway, a crucial protein degradation system, were also found to be significantly enriched.

**Conclusions:**

The in-depth proteomic characterisation of this large-scale CKD cohort is a step toward generating mechanism-based hypotheses that might lend themselves to future drug targeting. Candidate biomarkers will be validated in samples from selected patients in other large non-dialysis CKD cohorts using a targeted mass spectrometric analysis.

**Supplementary Information:**

The online version contains supplementary material available at 10.1186/s12014-023-09405-0.

## Background

Chronic kidney disease (CKD), characterised by progressive damage to the kidney due to a variety of pro-inflammatory, pro-fibrotic and renal circulatory insults [1], is an increasing public health problem that affects a large proportion of the adult population worldwide [2]. The increasing incidence of CKD is mainly driven by the increase in the prevalence of obesity, diabetes mellitus, hypertension and ageing [3]. Although precise calculation of the burden is difficult due to the lack of symptoms until disease is advanced [4], recent studies have calculated the global prevalence of CKD to be 13.4% (95% confidence interval 11.7–15.1%) [5]. In 2017, CKD was estimated to be the 12th leading cause of death globally, causing around 1.2 million deaths that year [6]. This death toll and the large increase of patients with end-stage kidney disease (ESKD) needing renal replacement therapy (RRT), cause substantial financial burden for even the wealthiest countries [5]. In England, around 2% of the National Health Service’s budget is spent on the 0.1% of the population who have ESKD, and the overall cost of treating CKD was previously estimated to be £1.4 billion/year [7].

Due to the significant health burdens for patients and the costs incurred by already stressed health-care systems, halting or slowing progression of CKD towards ESKD is a major goal for clinicians, researchers, patient groups and industry worldwide. Mechanistic components are thought to include pro-fibrotic, pro-inflammatory, and vascular pathways, but the understanding of temporal pathophysiology is limited in most CKD aetiologies and current treatments are non-specific, with heterogeneity in terms of response and outcome.

In the past few decades, extensive research has been carried out to explore potential mechanisms responsible for the development of renal diseases and the progression of renal fibrosis and progressive nephron loss, but still, the underlying mechanisms of CKD progression are not fully understood. In most renal diseases, differentiation of patients at risk of more rapid progression of renal dysfunction is limited to crude markers such as estimated glomerular filtration rate (eGFR) and albuminuria, as encapsulated in tools such as the Kidney Failure Risk Equation (KFRE). The identification of more precise progression biomarkers (especially if mechanistically important) would help target earlier treatments and reduce the burden of complications in patients with CKD.

In this study, we proposed to investigate whether proteomic signatures can be derived, since the development of biomarkers that associate with different rates of progression would transform the ability to trial emerging therapies such as novel anti-fibrotic agents, or mechanism-based anti-inflammatory agents, and thereby introduce new agents into clinical care.

## Materials and methods

### Study population

The Salford Kidney Study (SKS), is a longitudinal, prospectively collected, ongoing observational study with full ethical permission that has recruited and followed-up patients with non-dialysis dependent CKD (NDD-CKD) in the United Kingdom since March 2002 [25, 28]. This large NDD-CKD cohort consists of > 3500 patients that have provided their informed consent when admitted to the nephrology inpatient centre at Salford Royal Hospital or referred to nephrology outpatient clinics [8, 26]. The participants in the current study were ≥ 18 years at the time of consent with (eGFR) < 60 ml/min/1.73 m^2^ who had not started renal replacement therapy (RRT). Detailed phenotypic, co-morbidity and laboratory data is collected for each patient at baseline and at annual review in routine clinic visits. Patients are followed until discharge, death, or withdrawal from the study [8, 27]. At each visit, samples including EDTA whole blood, serum and citrate plasma were collected, centrifuged and bio-banked at  −80 °C in the local Biological Repository for future biomarker and genomic research.

### GFR slope calculation and patient selection

Serum creatinine at routine clinic visits was measured using a calibrated Jaffe method traceable to an isotope dilution mass spectrometry reference measurement procedure. This permits the GFR to be estimated using the CKD-EPI equation, and CKD-EPI eGFR values were used to calculate GFR slopes (∂ eGFR) in this study. Calculation of the ∂ eGFR for each patient involved use of ordinary least-squares linear regression to all outpatient eGFR values during study follow-up; only patients with at least 4 eGFR values over 2 years were included in the study [9].

Patients were defined as having fast progression if a ∂ eGFR of < − 3 ml/min/1.73 m^2^/year (in other words, losing more than 3 ml/min/1.73 m^2^/year) was observed. Stable patients were defined as having a ∂ eGFR of − 0.5 to + 1 ml/min/1.73 m^2^/year [10]. In this study only patients with a linear form of progression (consistent decline of eGFR slope) were included. To achieve this, each patient's eGFR-time slopes were visually reviewed by two researchers independently, a methodology that has been employed before [8]. In addition, the 95% confidence interval was also calculated for each patient's delta GFR. The smaller the interval, the greater the degree of a consistent linear pattern, and this provided a quantitative measure of eGFR linearity [8]. Patients were included from several renal disease groups: diabetic nephropathy, hypertensive nephropathy, autosomal dominant polycystic kidney disease (ADPKD), glomerulonephritis, but also those patients with ‘other’ CKD and unknown cause of CKD.

### Sequential window acquisition of all theoretical fragment ion spectra (SWATH) analysis

Plasma samples were prepared as described by KA [11]. Briefly, major plasma proteins were removed using Top 12 Abundant Protein Depletion Spin columns (Pierce Biotechnology, UK). Amicon Ultra-0.5 Centrifugal Filter Devices (Merck-Millipore, UK) were used to concentrate the eluate and for buffer exchange. Protein concentration was measured using a BCA protein assay kit (Thermo Fisher, UK) with a multi-mode plate reader Spectramax i3 (Molecular Devices, UK) at 562 nm wavelength. The depleted plasma was denatured, reduced and alkylated in 25 mM ammonium bicarbonate containing 5 mM dithiothreitol (GE Healthcare, UK), 50 mM iodoacetamide (Sigma Aldrich, UK) and 1% sodium deoxycholate (Sigma Aldrich, UK). Modified sequencing-grade trypsin (Promega, UK) was added and digestion was performed overnight at 37 °C. The samples were concentrated using a MiVac vacuum centrifuge GenevacTM (Thermo Fisher Scientific, UK). Lyophilised peptide samples were reconstituted in loading buffer containing 2% (v/v) acetonitrile, 0.1% (v/v) formic acid, 100 fmol/μl PepCalMix (MS Synthetic Peptide Calibration Kit, AB Sciex UK Ltd, UK) and 10 × index retention time (iRT) standards (Biognosys AG, Switzerland). After reconstitution, 10 μl of sample (containing 8 µg of total protein) was injected for chromatographic separation. In parallel with individual study samples, a pool of study samples and a commercially sourced plasma sample (BioIVT, UK) were processed to access efficiency in depletion/digestion.

Samples were analysed by SWATH-MS with a micro-flow LC–MS system comprising an Eksigent nanoLC 400 autosampler and an Eksigent nanoLC 425 pump coupled to a AB Sciex 6600 Triple-TOF mass spectrometer with a DuoSpray Ion Source. The system was configured for trap-elute analysis in which sample was injected from the autosampler (8 °C) onto a trap column (YMC-Triart C18; length: 5 mm; ID: 0.5 mm; particle size: 3 µm; pore size: 120 Å) with loading buffer mobile phase (10 μl min^−1^, 3 min, 2% acetonitrile, 0.1% formic acid) then eluted through an analytical column (YMC-Triart C18; length: 150 mm; ID: 0.3 mm; particle size: 3 µm; pore size: 120 Å; 30 °C) with the required analytical gradient into the mass spectrometer source. The system was controlled by Analyst software v1.7.1 and Eksigent control software v4.2 (AB Sciex, The Netherlands).

Reverse-phase chromatography was performed at 30 °C with a flow rate of 5 μl/min over a 120-min gradient. Mobile phase A contained 100% LC/MS water with 0.1% (v/v) formic acid and mobile phase B contained 100% acetonitrile with 0.1% (v/v) formic acid. Samples were run as duplicate injections with blanks between each sample. For SWATH-MS analysis samples were eluted with an analytical gradient (3–40% acetonitrile, 0.1% formic acid) and a mass spectrometry method with a total duty cycle of 2.8 s comprising a TOF MS1 scan that was acquired over the mass range (m/z) 400–1250 followed by 100 SWATH-MS scans (m/z 100–1500) with variable m/z isolation widths, collision energy and collision energy spread. The voltage of spray was set at 5500 V. Mass spectrometry compatible K562 human protein extract digest (Promega, UK), pooled study sample and commercial plasma digest were used to monitor instrument performance during the study. SWATH-MS analysis was performed with specific mass spectrometric conditions, including isolation window size, overlap and total cycle time [29], that enabled protein-relative quantification of over 900 proteins. SWATH-MS data was searched using openSWATH (v2.0.0) against a large plasma spectral library (generated at Stoller Centre) with peptide matches scored using pyProphet (v0.18.3) and search results aligned using the TRIC algorithm from MSproteomicstools. Downstream analysis was performed in R (version 3.5) with transitions filtered based on m-score, as determined by pyProphet, at a threshold of 0.01. The Bioconductor (release 3.5) packages SWATH2Stats and MSstats were used for summarisation and normalisation, with a minimum of one proteotypic peptide per protein required for quantification.

The resulting SWATH map was investigated with reference to clinical data in order to identify potential blood-borne biomarkers of rapid CKD progression versus stable CKD. Proteins present in at least 20% of the samples were retained for the biomarker analysis in our study.

### Statistical and data analysis

Statistical tests and downstream analysis using machine learning approaches for discovery (Random Forest and Boruta Feature Selection) were performed using the computing environment R (version 4.1.0) and additional software packages including limma, Biobase and UniprotR were obtained via the Bioconductor project (packages release 3.13). Proteomic data was log_2_ transformed to stabilise the variance and reduce heteroscedasticity. Negative values in initial data (resulting from log transformation of values < 1) were omitted from the analyses and missing values were replaced by zeros. For hypothesis testing, the t-test was used to identify differentially expressed proteins between the two patient groups (a *p*-value < 0.05, after multiple testing corrections was considered statistically significant). Fold changes between our stable CKD and rapid progressor groups were calculated for each protein using the ‘limma’ package in R (Bioconductor packages release 3.13). The caret package (version 6.0.89) and a random seed of 89 was used to create an index with 65% of data to create a training set and stratify the partition by the progressor type. Feature selection was undertaken using the Boruta algorithm [30], which has recently been applied to SWATH-MS data [12] and has shown to be effective in permutation-based feature selection [13]. The Boruta R package (version 7.0.0) was deployed with a random seed of 734, the parameter nt*ree* set to 500 and the parameter *maxRuns*, which specifies maximum runs that the algorithm will iterate, set to 4000. After feature selection made by the Boruta algorithm, a random forest model was built. Accuracy was used to select the optimal model using the largest value. The cumulative AUC for the addition of each biomarker, in order of its Boruta importance, was calculated using the Cstat function from the DescTools package (version 0.99.43).

Enrichment testing using the list of potential biomarkers identified by the Boruta algorithm was performed using the Database for Annotation, Visualisation and Integrated Discovery (DAVID version 6.8) and ClueGo (version 2.5.7), a plug-in feature in Cytoscape (version 3.8.2). The following databases were used: GO Biological Process, GO Molecular Functions, GO Immune System Process, KEGG pathways, Reactome Pathways, and Wiki Pathways. Only pathways or functions with adjusted *p*-value < 0.05 (calculated using a two-sided hypergeometric test and Bonferroni step down correction) and a minimum of two proteins per pathway were considered. Pathway enrichment/depletion analysis was undertaken in a two-sided hypergeometric test using Bonferroni step down correction, and a minimum level of 4 was used for GO Tree Interval and minimum 2 genes per GO Term/Pathway selection. The list of biomarkers identified by RF were also used to run the DAVID functional annotation tool. The default Human gene list was used as background. The biological and molecular relevance of each predictor was estimated statistically and was adjusted for multiple-testing correction by Benjamini–Hochberg procedure.

## Results

### Demographic information

The population for our study consisted of 414 patients with a broad range of kidney disease aetiologies (Table 1) from the SKS cohort that were divided into 170 fast progressors and 244 stable patients using the eGFR slope analysis described in the “Materials and methods” section.

**Table 1:** Clinical profile of patients enrolled in the study

	Rapid progressors	Stable patients
Number of patients	170	244
Age mean, years	55.6	65.2
Age median (with interquartile ranges), years	54.7 (46.1–66.0)	66.9 (56.7–76.5)
Male gender, n (%)	92 (54)	167 (68)
White, n (%)	159 (94)	238 (98)
Systolic blood pressure median (with IQR), mmHg	142 (130–152)	136 (122–147)
Diastolic blood pressure median (with IQR), mmHg	80 (71–88)	73 (66–81)
Hypertension, n (%)	162 (95)	228 (93)
Diabetes, n (%)	49 (29)	82 (34)
Smoking, n (%)	105 (62)	153 (63)
Myocardial infarction, n (%)	8 (5)	22 (9)
Heart failure, n (%)	7 (4)	12 (5)
Stroke, n (%)	7 (4)	8 (3)
Peripheral vascular disease, n (%)	8 (5)	12 (5)
ACEi/ARB, n (%)	129 (76)	163 (67)
Statin, n (%)	101 (59)	165 (68)
Years follow-up median (with IQR)	4.8 (3.6–6.3)	6.5 (5.0–9.1)
*Primary renal disease*		
Diabetic nephropathy, n (%)	37 (22)	46 (19)
ADPKD, n (%)	43 (25)	4 (2)
Hypertensive nephropathy, n (%)	15 (9)	24 (10)
Glomerulonephritis, n (%)	23 (14)	42 (17)
Other, n (%)	39 (23)	89 (36)
Unknown, n (%)	13 (8)	39 (16)
*Laboratory results—median (with IQR)*		
eGFR (ml/min/1.73 m^2^)	32 (23–41)	25 (18–33)
ΔGFR (ml/min/1.73 m^2^/year)	− 4.75 (− 6.33 to − 3.70)	0.09 (− 0.24 to + 0.50)
eGFR results per patient, n	26 (17–37)	24 (16–40)
Bicarbonate (mmol/l)	22.0 (20.1–24.0)	22.8 (20.8–24.7)
Haemoglobin (g/l)	121 (113–131)	127 (117–137)
Calcium (mmol/l)	2.29 (2.19–2.38)	2.28 (2.20–2.36)
Phosphate (mmol/l)	1.14 (1.01–1.27)	1.06 (0.93–1.21)
Albumin (g/l)	42 (40–45)	44 (42–46)
uCPR (g/mol)	85 (22–294)	18 (9–38)
*Outcomes*		
ESRD, n (%)	111 (65)	24 (10)
Death prior to ESRD, n (%)	18 (11)	66 (27)

414 patients with a broad range of kidney disease aetiologies (including diabetic nephropathy, hypertensive nephropathy, autosomal dominant polycystic kidney disease, glomerulonephritis, other CKD and unknown cause CKD) were defined as having fast progression if a ∂ eGFR of < − 3 ml/min/1.73 m^2^/year (in other words, losing more than 3 ml/min/1.73 m^2^/year) was observed. Stable patients were defined as having a ∂ eGFR of − 0.5 to + 1 ml/min/1.73 m^2^/year [10]

Most of the patients in the study were Caucasian (94% in the rapid progression group and 98% in the stable progression group), and with a *p*-value of 2.333E−10, there was a significant mean age difference between our rapid and stable patients (mean age of rapid patients = 55.6 years old vs. mean age of stable patients = 65.17, see Additional file 1: Fig. S1). As shown in Table 1, there were significant differences between rapid progressors and stable CKD in terms of ∂ eGFR (− 4.75 vs. + 0.09 ml/min/1.73 m^2^/year), urine PCR (85 vs. 18 g/mol), and eGFR at study entry (32 vs. 25 ml/min/1.73 m^2^/year) but number of eGFR values per patient (26 vs. 24) were similar. A higher proportion of rapid progressors compared to stable CKD patients developed ESKD (65% vs. 10%) but fewer died before ESKD (11% vs. 27%).

Clinical features such as age, baseline creatinine and eGFR measurements were not accurate predictors of CKD progression, and whilst urine protein:creatinine ratio (PCR) was confirmed to have good discriminatory ability for the progression group in our cohort (AUC = 0.813, sensitivity = 0.431, specificity = 0.938), we analysed the proteomic signals in the plasma samples of our patients with the objective of gaining new insights into the pathophysiological processes involved in CKD and its progression.

### A proteomic signature of CKD progression

We employed proteomics to quantify a total of 943 proteins that were identified in all 617 samples from 414 unique patients. After analysing the missing values in the dataset, 626 proteins were found to be measurable in at least 20% of the samples (consisting of 66.38% of the original data) and were used for downstream analysis.

**Fig. 1:**
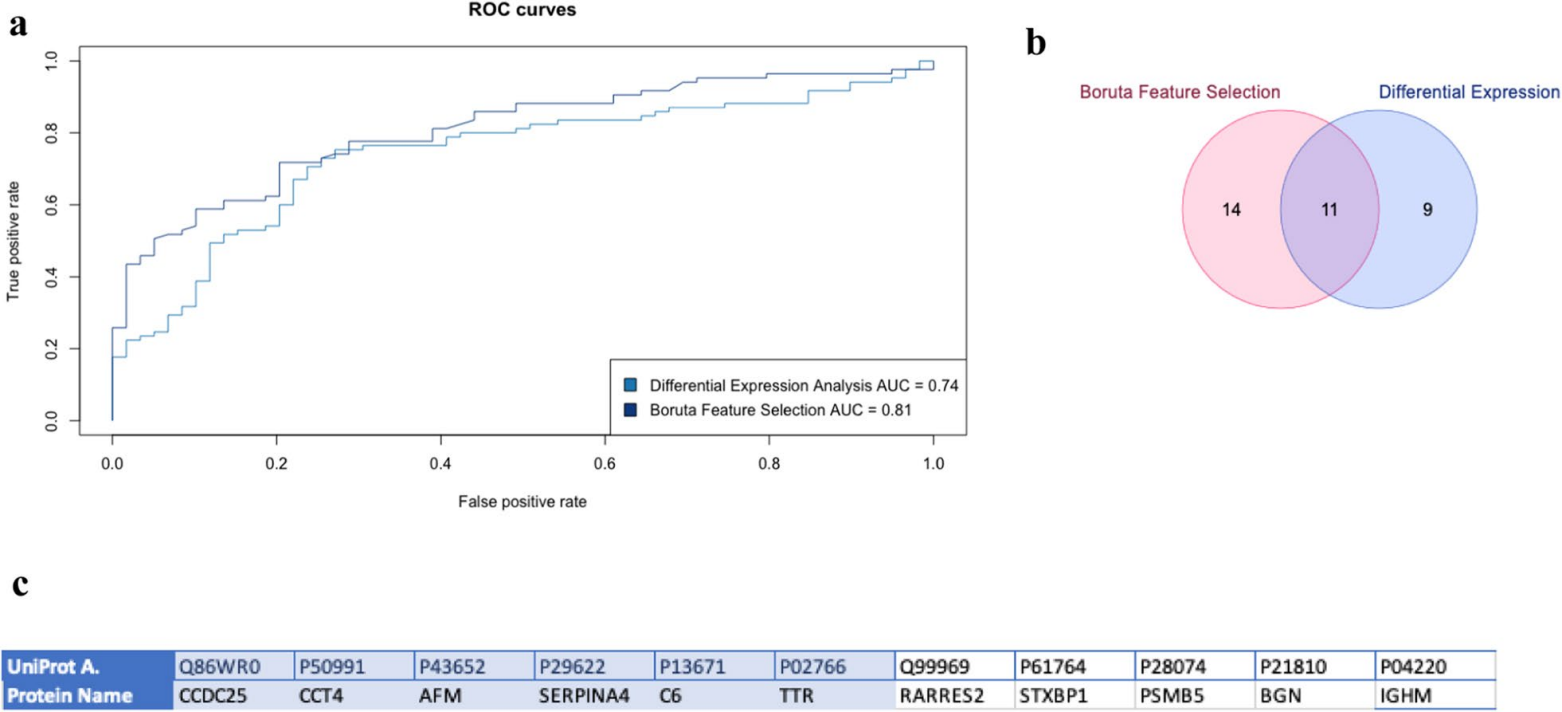
**a** ROC Curves showing the performance of the models built with the biomarkers identified by our Differential Expression Analysis and Random Forest with Boruta Feature Selection algorithm. The ROC curve for Boruta gives us the best AUC (0.81). **b** Overlap between the two sets of biomarkers (Differential Expression Analysis using limma and Random Forest model using Boruta Feature Selection. **c** Table with the 11 overlapping biomarkers coloured by significance (*p* value < 0.05) (Significant proteins: CCDC25, CCT4, AFM, SERPINA4, C6, TTR)

An initial differential expression analysis identified a total of 20 proteins (Additional file 1: Table S1) that exhibited significant differences between the rapid progressor and stable CKD groups (adjusted *p*-values < 0.05). Using the Boruta Feature Selection algorithm, 25 proteins were confirmed as important and are presented in order of their cumulative AUC in Table 2. Between these two approaches we found an overlap of 11 proteins which included: *Afamin (AFM), T-complex protein 1 subunit delta (CCT4), Complement component C6 (C6), Coiled-coil domain-containing protein 25 (CCDC25), Transthyretin (TTR), Syntaxin-binding protein 1 (STXBP1), Kallistatin (SERPINA4), Proteasome subunit beta type-5 (PSMB5), Biglycan (BGN), Immunoglobulin heavy constant mu (IGHM)* and *Retinoic acid receptor responder (RARRES2)*, see Fig. 1c.

**Table 2:** List of protein biomarkers identified using Boruta Feature Selection with their log2 fold change, median permutation importance; *p*-values and cumulative AUC shown

Protein	Name	Log2 fold change	Median importance	Differential expression *p*-value	Differential expression Adj *p*-value	Cumulative AUC
*P50991*	***CCT4***	*1.92*	*12.3*	< *0.001*	*0.008*	*0.67*
P43652	AFM	− 0.26	7.955	< 0.001	0.001	0.684
Q86WR0	CCDC25	2.349	8.078	0.001	0.043	0.688
P02766	TTR	− 0.309	7.403	< 0.001	0.002	0.701
Q01581	HMGCS1	0.128	7.071	0.734	0.908	0.704
*P13671*	***C6***	− *0.137*	*6.177*	*0.001*	*0.043*	*0.711*
Q8NC51	SERBP1	0.849	6.902	0.018	0.153	0.711
P04220	IGHM	2.28	5.791	< 0.001	0.007	0.746
Q99969	RARRES2	− 2.464	5.4	< 0.001	0.001	0.749
Q02790	FKBP4	− 1.389	4.687	0.004	0.074	0.759
*P28074*	***PSMB5***	− *2.63*	*4.624*	< *0.001*	*0.007*	*0.76*
P13667	PDIA4	0.784	4.575	0.035	0.199	0.762
*P08670*	***VIM***	− *1.91*	*4.236*	*0.004*	*0.076*	*0.765*
P21810	BGN	1.647	4.193	< 0.001	0.02	0.777
*P02745*	***C1QA***	− *0.175*	*4.155*	*0.003*	*0.07*	*0.78*
*P20851*	***C4BPB***	− *0.526*	*4.114*	*0.032*	*0.194*	*0.786*
*P40227*	***CCT6A***	*1.277*	*3.888*	*0.086*	*0.31*	*0.786*
P52566	ARHGDIB	0.555	3.656	0.18	0.458	0.787
Q15746	MYLK	− 1.492	3.709	0.004	0.07	0.787
P61764	STXBP1	− 2.397	3.52	< 0.001	0.02	0.793
*P20618*	***PSMB1***	*0.17*	*3.417*	*0.825*	*0.955*	*0.794*
Q96HR3	MED30	0.524	3.33	0.123	0.369	0.795
P29622	SERPINA4	− 0.159	2.808	0.001	0.043	0.804
P22105	TNXB	0.284	3.05	0.371	0.642	0.806
P31943	HNRNPH1	1.624	2.99	0.014	0.135	0.806

The eight proteins that were found to be part of a specifical biological pathway after functional enrichment analysis have been italicized

In order to compare the predictive performance of the potential biomarkers identified by our two analysis approaches, a random forest model was generated with each of the two proteomic signatures. Figure 1a shows the Receiver Operating Characteristic (ROC) curves for each of our models. The machine learning classification model generated with the 25 potential biomarkers identified by the Boruta feature selection algorithm provided an Area Under the Curve value of 0.81, and with an accuracy of 0.72 (sensitivity = 0.63, specificity = 0.78, Fig. 1a).

**Fig. 2:**
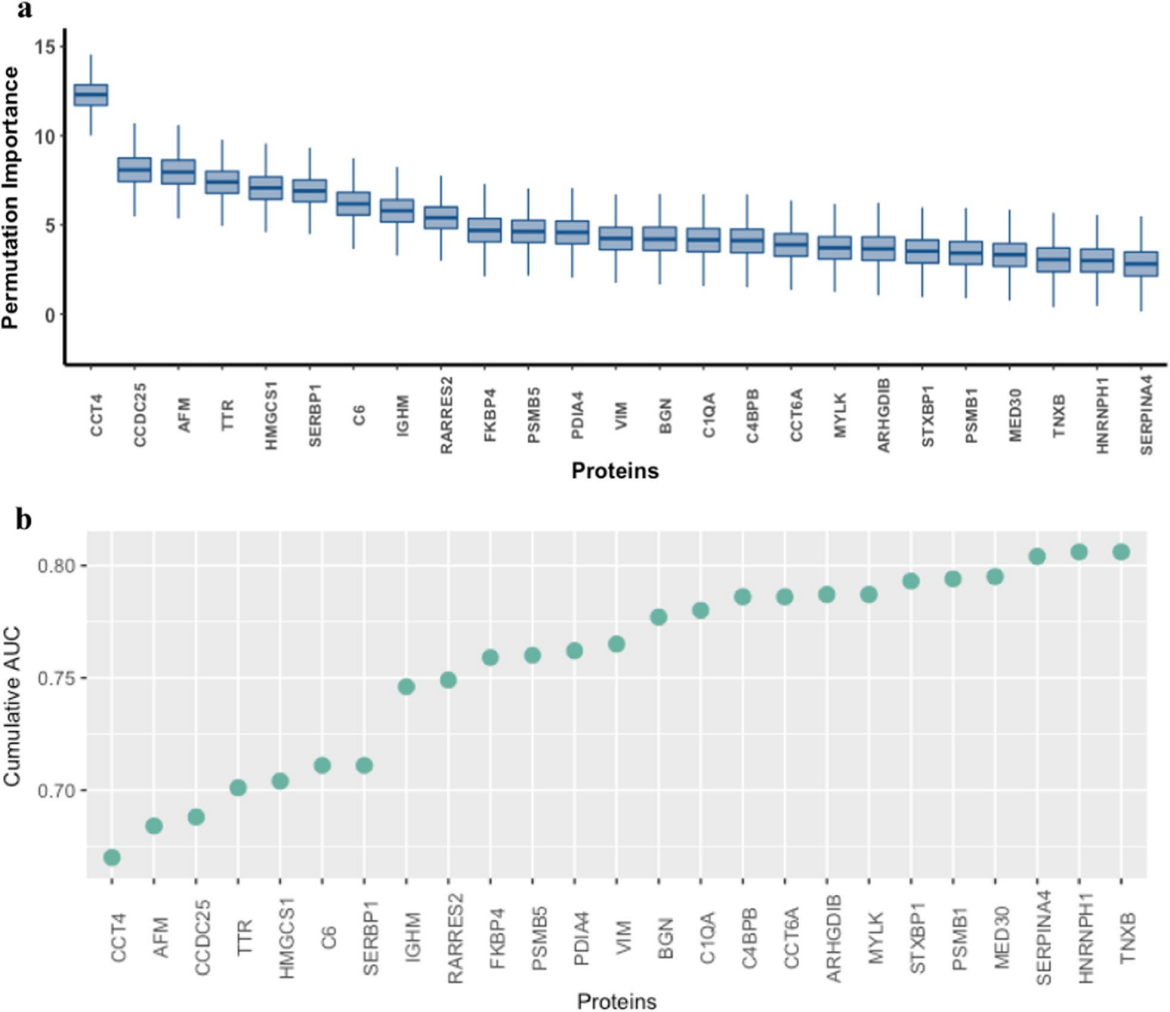
**a** Boxplot of Boruta importance among proteins found to be significant by the Boruta algorithm. **b** Cumulative AUC for Boruta-identified biomarkers calculated from logistic regression analysis

**Fig. 3:**
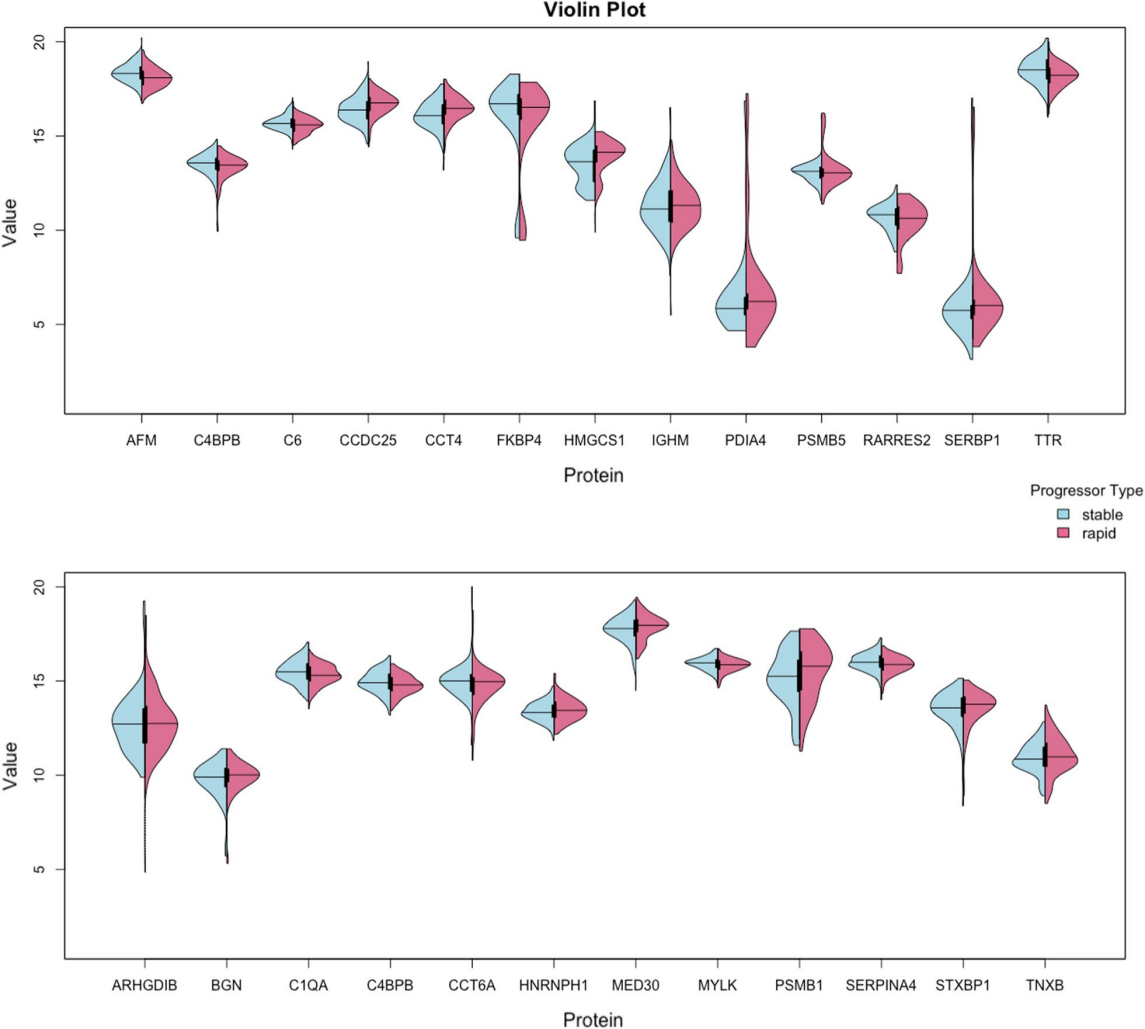
Violin plots. Distribution of the 25 confirmed proteins that form our core proteomic signature from Boruta Algorithm for our two progression groups

As an additional step, we also analysed how our 25-protein signature behaved within the top primary renal disease subgroups in our cohort. The first and main subgroup included any patient with diabetic nephropathy (type 1 and 2 DM) with the majority (90%) of the patients having DM type 2. Our second subgroup included different forms of glomerulonephritis (IgA Nephropathy, Membranous nephropathy and Focal segmental glomerulosclerosis). The rest of the primary renal disease aetiology subgroups were not included in the analysis due to class imbalance (e.g., in ADPKD, there were 43 progressors versus only 4 with stable CKD) and lack of sufficient data. In the two main subgroups, the best performance of our potential signature was obtained when used to predict the progressor type for the patients with diabetic kidney disease as the CKD aetiology (AUC = 0.68), and to a slightly lesser degree for the prediction of disease progression in the glomerulonephritis group (AUC = 0.67) (Figs. 2, 3).

### Functional enrichment analysis reveals role for complement and coagulation pathways

**Fig. 4:**
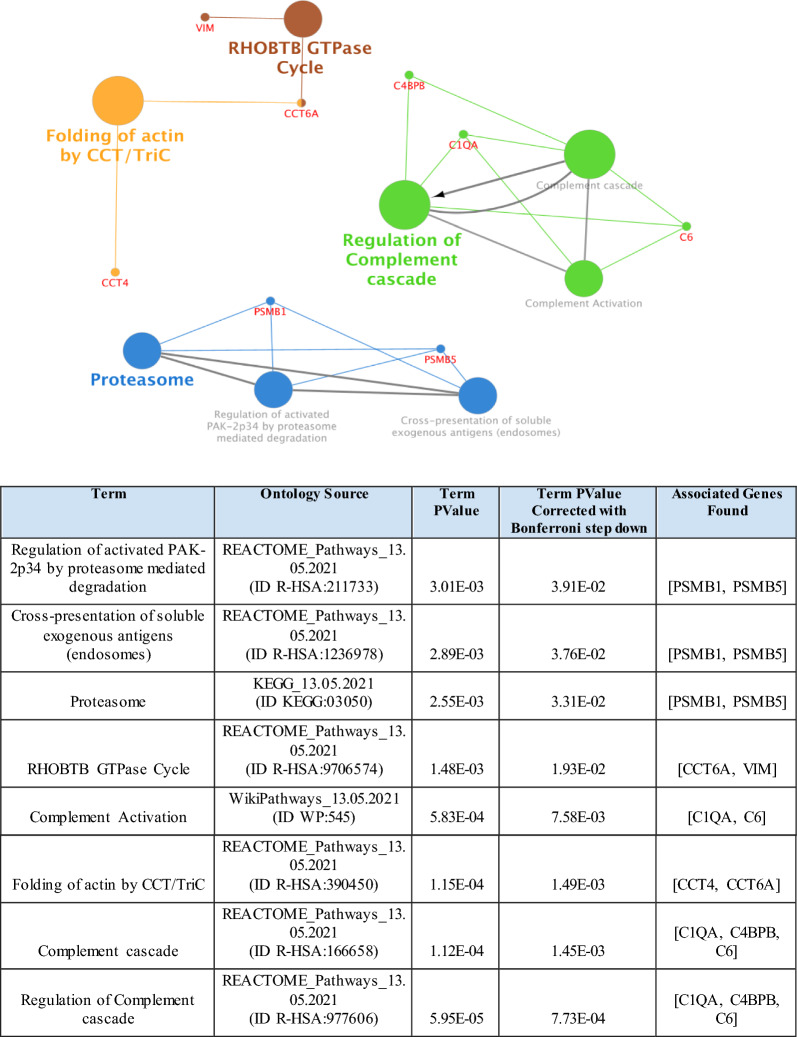
Functionally grouped networks of enriched pathways using tool ClueGo

In order to identify functional pathways associated with our proteomic signatures, and therefore identify mechanisms that may correlate with progression of CKD, pathway enrichment analysis was carried out. Statistically significantly enriched pathways identified by the Database for Annotation, Visualisation and Integrated Discovery (DAVID) and ClueGo functional enrichment conducted on the Boruta-identified proteins are presented in Additional file 1: Table S4. Both methods derived data that inferred the involvement of the complement cascade pathway as a putative pathogenic mediator of progressive renal disease; proteins in the complement cascade were significantly enriched (Term *p*-value corrected with Bonferroni step down = 1.45E−03). Using ClueGO tool and ontology source WikiPathways (13.05.2021) the proteasome pathway was also found to be statistically significant (Term *p*-value corrected with Bonferroni step down = 2.55E−03) (Fig. 4).

## Discussion

Current prediction of risk of CKD progression is crude, the most relevant traditional biomarker being albuminuria/proteinuria, which has limitations and provides minimal mechanistic insight. By looking at individual proteins that differentiate rapid progression from stable CKD new insights can be gained into the pathophysiological processes involved in deteriorating kidney function. This study has found some unique proteomic associations that differentiate CKD patients with more rapid progression from those with stable disease, importantly this is seen across a wide range of renal disease aetiologies. Although the differentiating proteins differ from those determined in other recent studies, the largest of which was by Grams et al. [14] that investigated CKD progression in the Atherosclerosis Risk in Communities Study (ARIC), this is not unexpected as the evaluated populations and study endpoints were not the same. In the latter, which was a study of almost 9500 people in the general US population (i.e., not primarily CKD) with median follow up of 14.4 years and of which 18% were self-reported black, the end points of the study were 50% decline in eGFR or ESKD development [14], differing from our endpoint of ∂ eGFR. In ARIC, the proteins associated with this renal endpoint were TNF receptor superfamily members 1A and 1B, trefoil factor 3, and b-trace protein, and these were then validated in two CKD cohorts, but the ethnic mix of these two US cohorts was markedly different to the SKS in which 96% of the patients were white.

Our study determined that the proteins which were differentially present in rapid CKD progression compared to those with stable eGFR were clustered mainly in the complement cascade and proteasome pathway. These findings are relevant since complement activation is known to occur in the kidney during the progression of many diverse renal diseases and could contribute to the chronic inflammation in which fibrosis occurs [15]. Impairment of proteasome function has also been reported to lead to CKD [16] and multiple studies have explored the role of intracellular protein degradation in CKD [17] (Additional file 2). 

We compared the predictive value of our proteomic signature and Urinary Protein-to-Creatinine Ratio. Our proteomic signature performs almost as good as uPCR when distinguishing the stable and the rapid progressors (AUC 0.79 vs. 0.81 respectively), but it is when we use both together that we get the best predictive performance (AUC 0.85).

### The complement cascade pathway and CKD

The complement cascade system, which consists of more than 30 proteins, serves as one of the first lines of defence for host protection against infection and for maintaining host hemostasis [31]. Although traditionally it has been regarded as an important part of the immune system, compelling evidence has shown that uncontrolled complement activation is also a pivotal pathogenic mediator of renal diseases in humans, and that it contributes to the damage that occurs during chronic renal progression through various mechanisms including direct pro-inflammatory and fibrogenic activity. It has been known for a long time that there is an association between the complement system and certain renal diseases. Uncontrolled complement activation leads to the generation of multiple effector compounds including C3a, C5a and MAC, which are detrimental to the host and contribute to progressive glomerulosclerosis and interstitial fibrosis.

There is growing evidence indicating that complement activation may contribute to the susceptibility and progression of diabetic nephropathy, the leading cause of CKD worldwide. However, further work is required to fully define the role of the complement system in clinical disease progression [15], since the presence of inflammation and upregulation of the renin–angiotensin–aldosterone system will also stimulate activation of the complement system.

There is a foundation for considering targeting complement as a potential approach to modulating the progression of diabetic nephropathy, and products that attenuate over activity of the complement system are now either available for use in nephrology (e.g. eculizamab) or under development. Inhibition of complement has the potential to abrogate disease progression and improve patient health. Nevertheless, a major effort is still required to understand the role of the complement pathway in the progressive loss of renal function, and to develop inhibitors that can be applied to treat more patients effectively in routine clinical practice.

### The ubiquitin–proteasome pathway and its association to CKD

The proteasome is a large multi-catalytic protease that degrades poly-ubiquitinated proteins to small peptides [18]. This protein-destroying apparatus plays a pivotal role in protein quality control [19] and involves many essential cellular functions, such as inflammatory responses, regulation of cell cycle, immune response, stress signalling, DNA repair and apoptosis [20]. In order to be degraded by the proteasome, proteins need to be tagged with Ubiquitin (Ub), a 76 amino acid regulatory protein found in nearly all eukaryotic organisms. The failure or dysregulation of the ubiquitin–proteasome pathway (UPP) prevents the degradation of misfolded proteins, leading to the disruption of normal cellular functions and even causing cell death [21]. Decreased proteasome function has been linked to many diseases such as immunological disorders, neurodegenerative [22] and cardiovascular diseases, cancer and inflammatory diseases. UPP has been identified to be significantly activated in patients with CKD [23], and it has been concluded that decreased proteasome function of podocytes leads to apoptosis, which results in CKD [16]. Our study provides further evidence of the relationship between proteasome impairment and CKD and highlights the importance of regulating a defective UPP in the treatment of CKD.

Nonetheless, this work is limited by the following considerations. The proteomic signatures were assessed in patients who had a wide range of eGFR, and it is possible that signals will vary according to the point in the CKD progression pathway of individual patients. Also, the proteomic findings only represented associations of rapid progression or stable CKD; demonstration of their mechanistic importance requires extensive further studies, including a consideration of complement pathway action in renal biopsy tissue acquired at different stages of the CKD progression [15]. The SKS population was also of predominantly white ethnicity and hence the generalisability of our proteomic findings to other cohorts remains to be determined. Some of these limitations can be addressed by replicating the methodology in further cohorts with more diverse ethnic backgrounds and focusing on differences between patients at different stages of the CKD pathway.

Our study of CKD progression is exploratory and limited to plasma proteomic profiling. Previous studies of renal disease have used different approaches including analysis of proteomics in plasma only, urine only, or both [24]. Although urinary proteomic analysis would include proteins released from damaged nephrons (glomeruli and tubules), ongoing processes in the interstitium and other areas of the kidney might not be properly captured, hence our decision to focus only on plasma analysis.

Additionally, although we have performed internal validation on test data, our machine learning models developed here remain to be validated in independent external samples, something that would need to be addressed in future work. Nevertheless, the in-depth proteomic characterisation of this large-scale CKD cohort is a step forward in generating mechanism-based hypotheses that might then lend themselves to future drug targeting. Candidate proteomic biomarkers will be validated in samples from selected patients in other large CKD cohorts using a targeted mass spectrometric analysis.

### Supplementary Information

**Additional file 1. Fig. S1**: Box plot comparison between rapid and stable patients and their mean age. **Fig. S2**: ROC Curves for the model built to test performance of our potential biomarkers in patient primary disease cause subgroups (Diabetes and Glomerulonephritis). **Fig. S3**: Box plot comparison between rapid and stable patients and the levels of Complement 6 protein. **Fig. S4**: Volcano Plot showing differentially expressed proteins. **Table S1**: Differential expression analysis results. **Table S2**: Primary disease causes code and number of patients. **Table S3**: *p*-values of *T*-test to determine if there is a significant difference between the means of our two progression type groups. **Table S4**: Functional Annotation Chart for enrichment analysis with the Database for Annotation, Visualisation and Integrated Discovery (DAVID).

**Additional file 2.** Log2 Proteinidentification and quantification data for all individual samples

## Data Availability

The datasets used and/or analysed during the current study are available from the corresponding author on request; these, as well as scripts used for analysis are also available from https://github.com/carlosramirezmedina/CKD_Proteomics_Data_Analysis. All protein identification and log2 protein quantification data for all individual samples can be found in Supplementary File #2.

## Abbreviations

ACEi: Angiotensin-converting enzyme inhibitors
ADPKD: Autosomal Dominant Polycystic Kidney Disease
ARB: Angiotensin receptor blockers
AUC: Area Under the Curve
CKD: Chronic Kidney Disease
DM: Diabetes Mellitus
eGFR: Estimated glomerular filtration rate
ESKD: End Stage Kidney Disease
KFRE: Kidney Failure Risk Equation
NDD-CKD: Non-dialysis Dependent CKD
PCR: Protein-Creatinine Ratio
RRT: Renal replacement therapy
SKS: Salford Kidney Study
SWATH-MS: Sequential Window Acquisition of all Theoretical fragment ion Mass Spectra
