# Correction: Proteomic signature associated with chronic kidney disease (CKD) progression identified by data-independent acquisition mass spectrometry

**DOI:** 10.1186/s12014-024-09471-y

**Published:** 2024-03-28

**Authors:** Carlos R. Ramírez Medina, Ibrahim Ali, Ivona Baricevic-Jones, Aghogho Odudu, Moin A. Saleem, Anthony D. Whetton, Philip A. Kalra, Nophar Geifman

**Affiliations:** 1 Stoller Biomarker Discovery Centre, Faculty of Biology, Medicine and Health, The University of Manchester, Manchester, UK; 2 Salford Royal Hospital, Northern Care Alliance NHS Foundation Trust, Salford, UK; 3 Division of Cardiovascular Sciences, The University of Manchester, Manchester, UK; 4 Bristol Renal and Children’s Renal Unit, Bristol Medical School, University of Bristol, Bristol, UK; 5 School of Veterinary Medicine, Faculty of Health and Medical Sciences, University of Surrey, Guildford, GU2 7XH UK; 6 School of Health Sciences, Faculty of Health and Medical Sciences, University of Surrey, Guildford, GU2 7XH UK

**Correction to: Clinical Proteomics (2023) 20:19**

10.1186/s12014-023-09405-0

In this article the affiliation details for authors Ivona Baricevic-Jones and Philip A Kalra were incorrectly given as “School of Veterinary Medicine, Faculty of Health and Medical Sciences, University of Surrey, Guildford GU2 7XH, UK” but should have been “Salford Royal Hospital, Northern Care Alliance Foundation Trust, Salford, UK”.

The original article has been corrected.

## References

[1] Ram?rez Medina C.R., Ali I., Baricevic-Jones I, et al. Proteomic signature associated with chronic kidney disease (CKD) progression identified by data-independent acquisition mass spectrometry. Clin Proteom. 2023;20:19. 10.1186/s12014-023-09405-0 10.1186/s12014-023-09405-0 PMC10116780 37076799

