# Supplementary material for: Proteomic signature associated with chronic kidney disease (CKD) progression identified by data-independent acquisition mass spectrometry
Source: Clin Proteomics. 2023 Apr 20;20:19. doi: 10.1186/s12014-023-09405-0 (PMC10116780; doi:10.1186/s12014-023-09405-0)
Supplement: Supplementary file 1 — Additional file 1. Fig. S1: Box plot comparison between rapid and stable patients and their mean age. Fig. S2: ROC Curves for the model built to test performance of our potential biomarkers in patient primary disease cause subgroups (Diabetes and Glomerulonephritis). Fig. S3: Box plot comparison between rapid and stable patients and the levels of Complement 6 protein. Fig. S4: Volcano Plot showing differentially expressed proteins. Table S1: Differential expression analysis results. Table S2: Primary disease causes code and number of patients. Table S3: p-values of T-test to determine if there is a significant difference between the means of our two progression type groups. Table S4: Functional Annotation Chart for enrichment analysis with the Database for Annotation, Visualisation and Integrated Discovery (DAVID). [file 12014_2023_9405_MOESM1_ESM.docx]

***Supplemental Material***


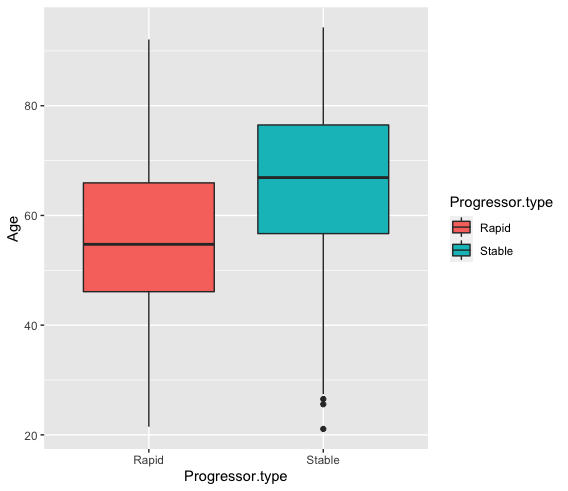


***Supplementary Figure 1*.** Box plot comparison between rapid and stable patients and their mean age.


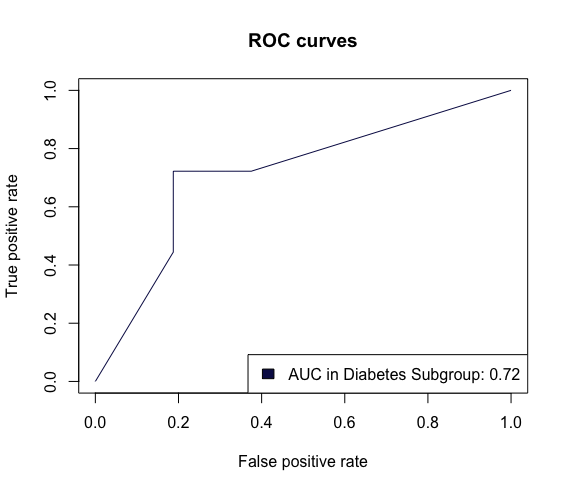

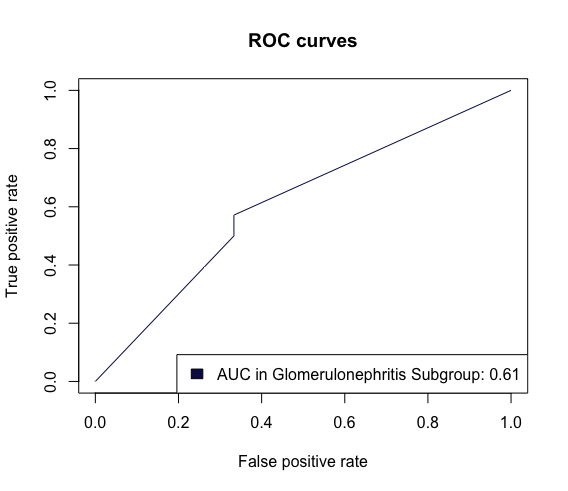


***Supplementary Figure 2*.** ROC Curves for the model built to test performance of our potential biomarkers in patient primary disease cause subgroups (Diabetes and Glomerulonephritis).


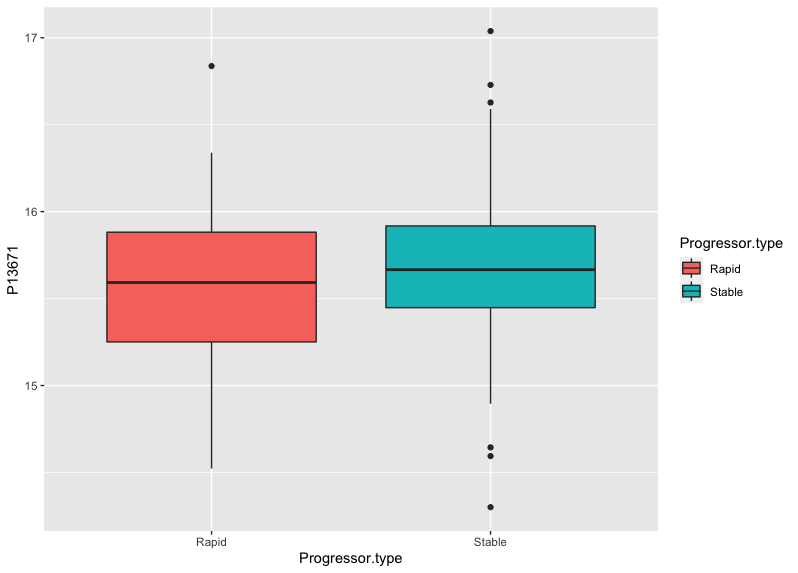


***Supplementary Figure 3.*** Box plot comparison between rapid and stable patients and the levels of Complement 6 protein.


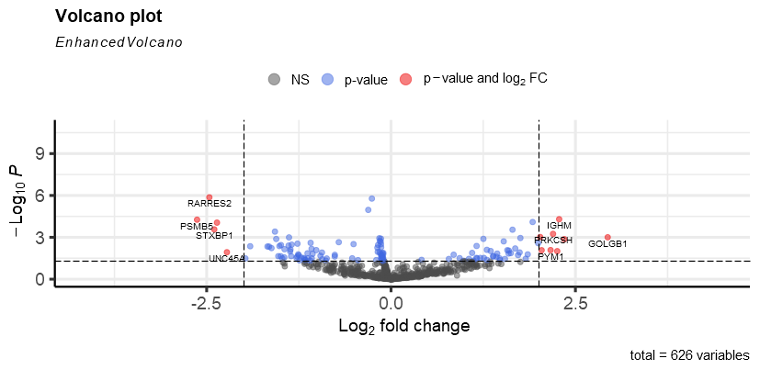


***Supplementary Figure 4*.** Volcano Plot showing differentially expressed proteins.

***Supplementary Table 1*.** Differential Expression Analysis Results

| **Uniprot ID** | **Name** | **Protein Name** | **logFC** | **AveExpr** | **P.Value** | **adj.P.Val** |
| --- | --- | --- | --- | --- | --- | --- |
| **P00746** | CFD | Complement factor D (EC 3.4.21.46) (Adipsin) (C3 convertase activator) (Properdin factor D) | 1.25 | 13.76 | 0 | 0.04 |
| **P02766** | TTR | Transthyretin (ATTR) (Prealbumin) (TBPA) | -0.31 | 18.37 | 0 | 0 |
| **P13671** | C6 | Complement component C6 | -0.14 | 15.64 | 0 | 0.04 |
| **P14314** | PRKCSH | Glucosidase 2 subunit beta (80K-H protein) (Glucosidase II subunit beta) (Protein kinase C substrate 60.1 kDa protein heavy chain) (PKCSH) | 2.2 | 8.22 | 0 | 0.03 |
| **P29622** | SERPINA4 | Kallistatin (Kallikrein inhibitor) (Peptidase inhibitor 4) (PI-4) (Serpin A4) | -0.16 | 15.94 | 0 | 0.04 |
| **P31327** | CPS1 | Carbamoyl-phosphate synthase [ammonia], mitochondrial (EC 6.3.4.16) (Carbamoyl-phosphate synthetase I) (CPSase I) | 1.75 | 12.19 | 0 | 0.04 |
| **P32119** | PRDX2 | Peroxiredoxin-2 (EC 1.11.1.24) (Natural killer cell-enhancing factor B) (NKEF-B) (PRP) (Thiol-specific antioxidant protein) (TSA) (Thioredoxin peroxidase 1) (Thioredoxin-dependent peroxide reductase 1) (Thioredoxin-dependent peroxiredoxin 2) | -1.56 | 7.61 | 0 | 0.04 |
| **P43121** | MCAM | Cell surface glycoprotein MUC18 (Cell surface glycoprotein P1H12) (Melanoma cell adhesion molecule) (Melanoma-associated antigen A32) (Melanoma-associated antigen MUC18) (S-endo 1 endothelial-associated antigen) (CD antigen CD146) | -1.38 | 6.95 | 0 | 0.04 |
| **P43652** | AFM | Afamin (Alpha-albumin) (Alpha-Alb) | -0.26 | 18.25 | 0 | 0 |
| **P04220** | IGHM | Immunoglobulin mu heavy chain | 2.28 | 6.29 | 0 | 0.01 |
| **P21810** | BGN | Biglycan (Bone/cartilage proteoglycan I) (PG-S1) | 1.65 | 3.04 | 0 | 0.02 |
| **P50991** | CCT4 | T-complex protein 1 subunit delta (TCP-1-delta) (CCT-delta) (Stimulator of TAR RNA-binding) | 1.92 | 14.66 | 0 | 0.01 |
| **P61764** | STXBP1 | Syntaxin-binding protein 1 (MUNC18-1) (N-Sec1) (Protein unc-18 homolog 1) (Unc18-1) (Protein unc-18 homolog A) (Unc-18A) (p67) | -2.4 | 8.25 | 0 | 0.02 |
| **Q86WR0** | CCDC25 | Coiled-coil domain-containing protein 25 | 2.35 | 12.07 | 0 | 0.04 |
| **P28074** | PSMB5 | Proteasome subunit beta type-5 (EC 3.4.25.1) (Macropain epsilon chain) (Multicatalytic endopeptidase complex epsilon chain) (Proteasome chain 6) (Proteasome epsilon chain) (Proteasome subunit MB1) (Proteasome subunit X) | -2.63 | 6.62 | 0 | 0.01 |
| **Q99969** | RARRES2 | Retinoic acid receptor responder protein 2 (Chemerin) (RAR-responsive protein TIG2) (Tazarotene-induced gene 2 protein) | -2.46 | 3.98 | 0 | 0 |
| **Q9H0W9** | C11orf54 | Ester hydrolase C11orf54 (EC 3.1) | -2.36 | 11.34 | 0 | 0.01 |
| **O00203** | AP3B1 | AP-3 complex subunit beta-1 (Adaptor protein complex AP-3 subunit beta-1) (Adaptor-related protein complex 3 subunit beta-1) (Beta-3A-adaptin) (Clathrin assembly protein complex 3 beta-1 large chain) | 2.02 | 4.68 | 0 | 0.04 |
| **Q14789** | GOLGB1 | Golgin subfamily B member 1 (372 kDa Golgi complex-associated protein) (GCP372) (Giantin) (Macrogolgin) | 2.94 | 5.69 | 0 | 0.04 |
| **Q14515** | SPARCL1 | SPARC-like protein 1 (High endothelial venule protein) (Hevin) (MAST 9) | -1.57 | 2.76 | 0 | 0.02 |

***Supplementary Table 2*.** Primary Disease Causes Code and Number of patients.

| **Code** | **Occurrences** | **Disease** | **Rapid** | **Stable** |
| --- | --- | --- | --- | --- |
| **80** | **83** | **Diabetes Type 1 and 2** | **37** | **46** |
| 1 | 52 | Aetiology Uncertain | 13 | 39 |
| 41 | 47 | Polycystic Kidneys - Adult Type (Dominant) | 43 | 4 |
| 72 | 39 | Renal Vascular Disease due to Hypertension | 15 | 24 |
| **12*** | **30 (12+ 18)** | **IgA Nephropathy (Proven by Immunofluorescence)** | **20** | **29** |
| **14*** | **10 ( 5 + 5)** | **Membranous nephropathy** |  |  |
| **17*** | **9 ( 3 + 6 )** | **Focal segmental glomerulosclerosis with nephrotic syndrome in adults** |  |  |

***Supplementary Table 3.*** P-values of T-test to determine if there is a significant difference between the means of our two progression type groups.

| **Protein** | **Name** | **All Data** | **Diabetes Subgroup** | **Glomerulonephritis Subgroup** |
| --- | --- | --- | --- | --- |
| **P43652** | AFM | 1.17E-06 | 0.002137 | 0.1222 |
| **P50991** | CCT4 | 2.02E-06 | 0.01577 | 0.02467 |
| **P02766** | TTR | 4.34E-06 | 0.001264 | 0.8743 |
| **Q01581** | HMGCS1 | 3.41E-05 | 0.161 | 0.01355 |
| **Q86WR0** | CCDC25 | 0.000359 | 0.1188 | 0.3269 |
| **P30041** | PRDX6 | 0.000767 | 0.0003521 | 0.5016 |
| **P13671** | C6 | 0.000874 | 0.001816 | 0.1061 |
| **P02745** | C1QA | 0.002353 | 0.04061 | 0.01075 |
| **P01031** | C5 | 0.002964 | 0.03112 | 0.6577 |
| **P20851** | C4BPB | 0.003314 | 0.4325 | 0.0608 |
| **P61106** | RAB14 | 0.006154 | 0.099 | 0.2636 |
| **P02747** | C1QC | 0.007985 | 0.1605 | 0.01463 |
| **P09871** | C1S | 0.008341 | 0.209 | 0.254 |
| **Q9Y3I0** | RTCB | 0.01516 | 0.4865 | 0.2226 |
| **O00299** | CLIC1 | 0.02676 | 0.01966 | 0.4435 |
| **P40925** | MDH1 | 0.03221 | 0.03796 | 0.6193 |
| **P20618** | PSMB1 | 0.08305 | 0.3994 | 0.4333 |
| **P31943** | HNRNPH1 | 0.09314 | 0.8142 | 0.09947 |
| **P13667** | PDIA4 | 0.09562 | 0.499 | 0.2845 |
| **P08670** | VIM | 0.1001 | 0.313 | 0.6069 |
| **P28074** | PSMB5 | 0.1079 | 0.3993 | 0.5525 |
| **P19320** | VCAM1 | 0.2409 | 0.7077 | 0.188 |
| **Q99969** | RARRES2 | 0.2459 | 0.2912 | 0.9375 |
| **Q8NC51** | SERBP1 | 0.3168 | 0.7461 | 0.426 |
| **P07942** | LAMB1 | 0.4828 | 0.007224 | 0.3581 |
| **P21810** | BGN | 0.5134 | 0.7713 | 0.5041 |
| **P04220** | IGHM | 0.8837 | 0.9102 | 0.3836 |
| **P52566** | ARHGDIB | 0.9325 | 0.08171 | 0.3978 |

***Supplementary Table 4.*** Functional Annotation Chart for enrichment analysis with the Database for Annotation, Visualisation and Integrated Discovery (DAVID)

| ***Category*** | ***Term*** | ***Count*** | ***%*** | ***P-Value*** |
| --- | --- | --- | --- | --- |
| KEGG_PATHWAY | Complement and coagulation cascades | 3 | 12.5 | 0.0091 |
| KEGG_PATHWAY | Prion disease | 4 | 16.7 | 0.01 |
| KEGG_PATHWAY | Proteasome | 2 | 8.3 | 0.077 |
